# AI Self-Efficacy and Innovative Work Behavior in Hospitality and Tourism: A Job Demands-Resources Perspective on Work Engagement and Schedule I-Deals

**DOI:** 10.3390/bs16030431

**Published:** 2026-03-16

**Authors:** Xiaomeng Li, Ziyi Gong, Hyeran Choi, Seung-Wan Kang

**Affiliations:** 1 College of Business, Gachon University, Seongnam 13120, Republic of Korea; lixiaomeng1017@gachon.ac.kr; 2 College of Business & Economics, Chung-Ang University, Seoul 06974, Republic of Korea

**Keywords:** AI self-efficacy, innovative work behavior, work engagement, schedule idiosyncratic deals

## Abstract

As artificial intelligence becomes increasingly embedded in hospitality and tourism services, it is reshaping employees’ innovative work behavior. Grounded in the Job Demands-Resources perspective, this study examines how AI self-efficacy affects innovative work behavior and proposes a moderated mediation model to investigate the mediating role of work engagement and the boundary condition of schedule idiosyncratic deals. Using a three-wave time-lagged design, the study collected data from 300 employees working in the hospitality and tourism industry in Korea. The findings show that AI self-efficacy positively predicts innovative work behavior both directly and indirectly through increased work engagement. Furthermore, this mediating process is strengthened by higher levels of schedule i-deals, confirming a positive moderating effect. Theoretically, this study extends human-AI collaboration research by broadening the explanatory scope of the Job Demands-Resources model in the AI context. Practically, organizations undergoing digital transformation should provide training that strengthens employees’ confidence in using AI and grant greater autonomy over work schedules. Such practices help create a supportive environment that enables AI self-efficacy to translate into work engagement and ultimately innovative work behavior.

## 1. Introduction

The existence and development of service industries such as hospitality and tourism depend on meeting ever-changing customer service demands through innovation, making the enhancement of employees’ innovative work behavior a critical organizational objective (Ajmal et al., 2025; Aliane et al., 2023; Garg & Dhar, 2017). The increasing integration of artificial intelligence (AI) technologies into hospitality and tourism operations (Kong et al., 2025) raises an important question: Can the introduction of AI, then, stimulate employees’ innovative work behavior?

Prior research suggests a dual effect of AI on employees’ innovative work behavior (Yin et al., 2024). On the one hand, AI’s data-processing capabilities can help employees transcend the boundaries of knowledge and expand the scope of their thinking, thereby fostering innovation (Li et al., 2025). On the other hand, over-reliance on AI can diminish employees’ ability to think proactively and make independent decisions, potentially reducing cognitive flexibility and stifling innovative potential (J. Liu, 2025). These inconsistent findings highlight an important limitation in current AI research. Many previous studies focus mainly on AI’s effects on productivity or creativity (R. Wang et al., 2025; Zang & Li, 2025). However, they tend to overlook how employees’ individual differences, especially their beliefs in their capability to use AI, shape the actual process through which AI affects innovation. In other words, technology-centric approaches, while informative, are inherently limited in their ability to account for the heterogeneity of individual responses to AI—a gap that calls for a more person-centered theoretical lens. This technology-focused perspective often assumes that AI has the same impact on everyone, but it pays little attention to the role of human agency. Recent evidence shows that AI assistance does not influence all employees in the same way; rather, the creativity-enhancing effects of AI depend strongly on individual differences, with some employees able to leverage AI to generate more innovative ideas, while others gain little benefit or even struggle to adapt (Jia et al., 2024). Together, these findings suggest that AI technology alone does not determine employees’ innovative work behavior; rather, its effects depend substantially on individual characteristics.

Self-efficacy is a personal characteristic that promotes innovation and plays a significant role in shaping an individual’s beliefs and behaviors (Compeau & Higgins, 1995; Y.-Y. Wang & Chuang, 2024). Individuals with high self-efficacy voluntarily take on innovative tasks with high demands (Uppathampracha & Liu, 2022). In this study, employees’ AI self-efficacy is considered a precursor influencing innovative work behavior. Existing research has predominantly focused on self-efficacy in the use of traditional technologies, and studies on the role of self-efficacy in the AI domain remain limited (B.-J. Kim & Lee, 2025). Therefore, it is necessary to investigate the impact of AI self-efficacy on innovative work behavior more comprehensively.

AI self-efficacy refers to employees’ beliefs about their capability to use AI to perform work-related tasks (B.-J. Kim et al., 2024). In this study, AI refers to AI-powered digital technologies used to support work-related tasks and service delivery, including widely used applications in hospitality and tourism, such as chatbots, automated reservation systems, personalized recommendation systems, and intelligent automation tools (H. Kim et al., 2025; López-Naranjo et al., 2025). To comprehensively understand the impact of AI self-efficacy on innovative work behavior, this study, based on the Job Demands-Resources (JD-R) model, focuses on the psychological mechanisms and boundary conditions that shape the relationship between these two variables. This approach contributes to a clearer understanding of how and when its positive effects can be enhanced.

According to the JD-R model, personal resources refer to individuals’ positive self-evaluations that reflect their sense of control and capability to influence their work environment (Bakker & Demerouti, 2017). Self-efficacy is considered a key personal resource (Bakker & Demerouti, 2017), and an important antecedent of work engagement (Tims et al., 2011). Accordingly, AI self-efficacy can be understood as a personal resource that strengthens employees’ work engagement and thereby promotes innovative behavior. According to Ali et al. (2022), the influence of work engagement on innovative work behavior is inconsistent. Because the creative process is demanding and time-consuming (Zhang et al., 2018), employees may experience burnout when the necessary resources are not available (Kwon & Kim, 2020), which can hinder the transformation of work engagement into innovation. From this perspective, the extent to which work engagement translates into innovative behavior is likely to be influenced by work-related resources.

Schedule idiosyncratic deals (i-deals) are considered a job resource (Kelly et al., 2020) and refer to “an employee’s flexible daily work hours and focus on the employee’s ability to negotiate a flexible work schedule” (Velasco Vizcaíno et al., 2023, p. 3). This flexibility grants employees greater work autonomy (De Spiegelaere et al., 2016). Not only does it effectively alleviate the high-intensity work demands of the service industry (Kelly et al., 2020), but it also supports the accumulation of other resources (Van Waeyenberg et al., 2025). Furthermore, when leaders’ support interacts with employees’ motivation, it creates a more favorable environment for innovative work behavior (Venketsamy & Lew, 2022).

Although scholars generally agree that artificial intelligence is important for employees’ innovative work behavior, several research gaps remain. First, research on AI self-efficacy is still in its early stages and has primarily focused on the education sector (Bewersdorff et al., 2025; Morales-García et al., 2024). Consequently, few studies examine its impact on innovative work behavior in the hospitality and tourism industry. Second, existing AI-related research seldom integrates personal resources and work-related resources simultaneously within the JD-R model, limiting our understanding of human-AI collaboration (Y. Liu & Li, 2025). Finally, prior research has underexplored the psychological mechanisms and boundary conditions through which AI self-efficacy influences innovative work behavior. Although various studies (e.g., Aldabbas et al., 2023; Bernales-Turpo et al., 2022) have shown that work engagement plays a mediating role, its function in the AI context remains underexplored. In addition, previous research has primarily examined schedule i-deals as an independent or mediating variable (Taser et al., 2022; Velasco Vizcaíno et al., 2023). However, their moderating effect as an organizational resource has been overlooked. Taken together, these gaps highlight the need for an integrated JD-R perspective that simultaneously examines AI self-efficacy as a personal resource, work engagement as a motivational mechanism, and schedule i-deals as an organizational resource boundary condition in explaining employees’ innovative work behavior in AI-enabled service contexts.

Based on the above research gaps, this study constructs a moderated mediation model using the JD-R perspective to explore the mechanism through which AI self-efficacy promotes employees’ innovative work behavior. This study extends the existing literature on human-AI collaboration in several ways. First, by shifting from a technology-centric to a people-centric perspective and demonstrating the positive effect of AI self-efficacy on innovative work behavior, it provides new insights into understanding how service industry employees innovate in the AI era and responds to the call by Y.-Y. Wang and Chuang (2024) for more research on AI self-efficacy’s role in motivation and behavior. Second, by simultaneously considering the personal resource of AI self-efficacy and the job resource of schedule i-deals, this study expands the explanatory power of the JD-R model in the AI context. Third, by verifying the mediating effect of work engagement and the moderating effect of schedule i-deals, the study clarifies the mechanism linking AI self-efficacy to innovative work behavior. Finally, these findings suggest that organizations implementing AI technologies should strengthen employees’ AI self-efficacy, provide adequate support, and allow greater flexibility in work schedules to create conditions under which innovative motivation can translate into action.

## 2. Theoretical Background and Hypotheses

### 2.1. Relationship Between AI Self-Efficacy and Innovative Work Behavior

This study adopts the JD-R model (Bakker et al., 2014) as its theoretical foundation. The model emphasizes the role of job demands, personal resources, and work-related resources in shaping employee behavior and states (Demerouti & Bakker, 2011). The JD-R model has been widely applied to examine innovative work behavior in service industries such as hospitality and tourism (Bani-Melhem et al., 2020; Soliman et al., 2024).

Innovative work behavior is defined as “employees’ intentional efforts to generate, promote, and implement innovative ideas that benefit their work performance, the group, or the organization” (Schuh et al., 2018, p. 397). In service industries that rely heavily on service quality—such as hotels and tourism—innovative work behavior is essential for meeting diverse customer needs and enhancing organizational competitiveness (Ok & Lim, 2022). Moreover, within this sector, the effective implementation of innovative ideas is often more critical than idea generation alone (Kwon & Kim, 2020).

New initiatives inherently involve high uncertainty and intense demands (Choi et al., 2022). As artificial intelligence becomes increasingly widespread in the workplace, employees must continuously update their technical skills, and the use of AI itself has become a new job requirement (B.-J. Kim & Lee, 2024). According to the JD-R model, whether employees can maintain positive behavior under high-demand conditions is strongly influenced by personal resources (Bakker & Demerouti, 2007; Rabiul et al., 2022). Personal resources constitute forms of psychological capital that enable individuals to pursue goals and cope with stress (Bakker & Demerouti, 2017). Within the JD-R framework, AI self-efficacy can be viewed as a personal resource reflecting employees’ perceived capability to effectively cope with AI-related job demands.

As an emerging personal resource, AI self-efficacy encompasses not only the capacity to manage routine tasks with AI but also the ability to enhance problem-solving skills and innovative performance through its use (Morales-García et al., 2024). Employees with high AI self-efficacy are more likely to use AI tools effectively to streamline work processes, thereby reducing the time and effort required for repetitive tasks (Rick et al., 2024). Consequently, they can devote more resources to challenging and valuable innovative activities (H. Kim & Shim, 2025).

Additionally, innovative work behavior inherently involves risk, and employees’ willingness to engage in such behavior is influenced by their self-efficacy (Liang et al., 2023). When employees gain confidence in their AI abilities, their sense of control over complex tasks increases (Hong, 2022), and their anxiety and fear of failure when trying out new ideas decrease (B.-J. Kim et al., 2024). This sense of psychological safety and control derived from AI self-efficacy also enhances employees’ intrinsic motivation, increasing the likelihood that they translate innovative intentions into actual innovative work behavior. Such self-efficacy plays a crucial role in enabling employees to engage confidently in challenging tasks such as innovation and to successfully achieve related goals (Y. Liu et al., 2025). Past research in the hotel and tourism sector has similarly shown that individuals with high self-efficacy are more likely to display innovative work behavior (Teng et al., 2020; Wan et al., 2022). Therefore, based on the above reasoning, the following hypothesis is proposed:

**Hypothesis 1.**  *AI self-efficacy has a positive relationship with innovative work behavior, such that employees tend to demonstrate higher levels of innovative work behavior when they possess greater AI self-efficacy*.

### 2.2. Mediating Role of Work Engagement

A key question remains: Does AI self-efficacy lead directly to innovative work behavior, or does it first operate through employees’ motivational states to produce such outcomes? Employee work engagement is a key foundation for success in service industries such as hospitality and tourism (Rabiul & Yean, 2021). Because these industries rely heavily on high-quality service, organizations require highly engaged employees to meet diverse customer needs (Cheng & Chen, 2017). Work engagement has been defined as “a positive, fulfilling, work-related state of mind” (Schaufeli & Salanova, 2002, p. 74), characterized by vigor, dedication, and absorption (Bakker et al., 2014). Employees with high work engagement tend to exhibit strong positive attitudes and behavioral tendencies (Aldabbas et al., 2023; Memon et al., 2020). They respond more effectively to work demands, experience higher job satisfaction, and actively participate in innovative work behavior (Hassan et al., 2024; Schaufeli & Salanova, 2002). The JD-R model further suggests that highly engaged employees possess greater energy and are more responsive to changes in their work environment, thereby increasing the likelihood of innovative work behavior (Bakker et al., 2014).

According to the JD-R model (Bakker & Demerouti, 2007), personal resources serve as antecedents of work engagement (Mazzetti et al., 2023). Employees with high self-efficacy typically possess abundant psychological resources, enabling them to respond more proactively to job challenges and to exhibit strong coping abilities (Kong et al., 2025). AI self-efficacy is increasingly regarded as an essential personal resource in contemporary work environments (B.-J. Kim & Lee, 2024). When employees believe they can effectively use AI tools to improve work efficiency, this perceived sense of control fosters feelings of accomplishment and meaningfulness, thereby enhancing work engagement (Chen et al., 2022). When employees possess the necessary competencies and engage in their work with enthusiasm, innovative work behavior is more likely to occur (Jung et al., 2022).

According to the motivational process of the JD-R model, the influence of resources on work behavior is not always direct but is often manifested through the psychological mechanism of work engagement (Bakker et al., 2023). AI self-efficacy, as a personal resource, can promote innovative work behavior by enhancing employees’ levels of work engagement. Prior research has also demonstrated that work engagement mediates the relationship between self-efficacy and innovative work behavior (Uppathampracha & Liu, 2022). Thus, AI self-efficacy first energizes employees by helping them view their work as more meaningful and manageable, and this heightened motivational state then translates into the sustained effort required for innovative work behavior. Therefore, based on the above reasoning, the following hypothesis is proposed:

**Hypothesis 2.**  *Work engagement mediates the positive relationship between AI self-efficacy and innovative work behavior, such that employees with higher levels of AI self-efficacy tend to show greater work engagement, which subsequently leads to higher innovative work behavior*.

### 2.3. Moderating Role of Schedule I-Deals

Schedule i-deals are an important job resource, particularly in the hospitality and tourism industry, where employees commonly experience long working hours, high service pressure, and frequent customer interactions (Sun et al., 2023). These arrangements help employees achieve their work goals more effectively (Kelly et al., 2020), improve performance (Taser et al., 2022), and reduce work-family conflict, thereby promoting work–life balance (Erden Bayazit & Bayazit, 2019). Schedule i-deals constitute one type of i-deals (Las Heras et al., 2017b).

I-deals refer to “voluntary, personalized agreements of a nonstandard nature negotiated between individual employees and their employers regarding terms that benefit each party” (Rousseau et al., 2006, p. 978). Types of i-deals include schedule flexibility, location flexibility, job responsibilities, and financial incentives (Rosen et al., 2013). This study focuses specifically on schedule i-deals. Compared with other types of i-deals, schedule i-deals exert a stronger influence on employees’ temporal autonomy and work–life fit. These resources play a key role in transforming work engagement into innovative work behavior (Velasco Vizcaíno et al., 2023).

When employees receive high levels of schedule i-deals, it indicates that their managers have provided personalized job resources to address specific needs related to schedule allocation (Las Heras et al., 2017a). According to the JD-R perspective, adequate job resources help reduce the stress associated with high work demands (Bakker et al., 2023). These resources allow employees to allocate time and energy more effectively while accommodating task complexity and individual work patterns (Gajendran et al., 2015). Even under high levels of work engagement, temporal flexibility can alleviate scheduling-related stress, reduce energy depletion, and sustain cognitive vitality that supports innovative thinking (Avgoustaki & Cañibano, 2025). Under conditions where these resources are sufficiently available, work engagement can more smoothly translate into actual innovative work behavior. Consistent with this logic, prior research has shown that the greater the autonomy employees possess, the stronger the positive effect of work engagement on innovative work behavior (Garg & Dhar, 2017).

By contrast, employees who lack schedule i-deals must endure intense work demands within rigidly defined working hours. Even when such employees exhibit high levels of work engagement, their valuable energy and resources are more likely to be consumed by coping with time pressure, sacrificing personal time, or managing work–life conflict (Sun et al., 2023), thereby limiting the resources available for innovative thinking. Consequently, their engagement is less likely to translate into innovative work behavior. Conversely, schedule i-deals provide a protective buffer that preserves employees’ cognitive and temporal resources, enabling them to channel their motivational energy into concrete innovative actions and thereby reinforcing the positive effect of work engagement on innovation. Therefore, based on the above reasoning, the following hypothesis is proposed:

**Hypothesis 3.**  *Schedule i-deals strengthen the positive relationship between work engagement and innovative work behavior, such that employees with higher levels of schedule i-deals experience a stronger positive effect of work engagement on innovative work behavior*.

### 2.4. Moderated Mediation Mechanism of Schedule I-Deals

According to the JD-R model, personal resources promote positive behavioral outcomes primarily by enhancing work engagement as a motivational mechanism (Bakker & Demerouti, 2017). Thus, as a personal resource, AI self-efficacy fosters innovative work behavior through a motivational process that increases employees’ levels of work engagement. However, under high job demands, the motivational process linking personal resources to engagement may be accompanied by substantial physical and psychological resource depletion, potentially leading to burnout and diminishing the impact of work engagement (Yao et al., 2022). At this stage, job resources play a buffering role by mitigating the detrimental effects of high job demands (Bakker et al., 2014). These resources reduce employees’ resource depletion and enable personal resources to be more effectively translated into actual behaviors through motivation.

In the hospitality and tourism industries, employees face volatile and high-intensity work demands and must invest substantial time and energy in innovative initiatives (Barkat et al., 2024; Sumaneeva et al., 2021). To cope with these high demands, employees often seek additional resources to enhance work conditions and strengthen their ability to manage challenges (Bakker et al., 2023). Schedule i-deals represent personalized job-related resources that employees negotiate with their managers (Kelly et al., 2020). Employees who obtain such resources can adapt their work schedules and manage their time more flexibly (Sumaneeva et al., 2021), and in doing so, they also receive support from their managers (Van Waeyenberg et al., 2025).

Therefore, this study assumes that because schedule i-deals serve as a job resource that mitigates the negative impact of high work demands, the work engagement stimulated by AI self-efficacy is less likely to be rapidly depleted under such conditions. Instead, it is more likely to be sustained and to be converted into innovative work behavior. In other words, the motivational pathway from AI self-efficacy, through work engagement, to innovation operates most effectively when employees have the temporal flexibility to experiment and iterate—an advantage uniquely provided by schedule i-deals. Accordingly, the following hypothesis is proposed based on the above reasoning:

**Hypothesis 4.**  *Schedule i-deals moderate the mediating effect of work engagement on the relationship between AI self-efficacy and innovative work behavior, such that the positive indirect effect of AI self-efficacy on innovative work behavior through work engagement is stronger when employees have higher levels of schedule i-deals*.

**Figure 1:**
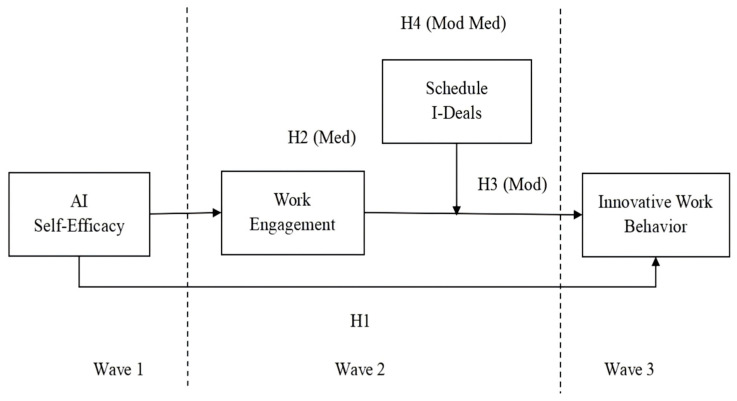
Research Model. Notes. Med = mediation; Mod = moderation; Mod Med = moderated mediation. Wave 1: AI self-efficacy. Wave 2 (one month after wave 1): work engagement and schedule i-deals. Wave 3 (one month after wave 2): innovative work behavior. Source: Authors’ own work.

Figure 1 presents the research model for all hypotheses.

## 3. Method

### 3.1. Research Design and Procedures

In an intensely competitive market environment, innovation serves not only as the foundation for achieving sustainable development but also as a core element for organizational survival and differentiation within service sectors (Aliane et al., 2023). Consequently, industries such as hospitality and tourism place strong emphasis on and actively encourage employees’ innovative work behavior (Jung et al., 2025). Given these industry characteristics, employees working in Korea’s hospitality and tourism sector were selected as the research sample.

Before conducting the study, formal approval was obtained from the university’s Institutional Review Board. Data were collected with the assistance of a professional online survey company, a subsidiary of a globally recognized marketing research organization. The company distributed and collected the questionnaires and matched participants across three survey waves using unique identifiers. The survey company maintains a nationwide panel of registered respondents who voluntarily enroll through online registration and verification procedures. Eligible participants were identified from this panel based on the researchers’ screening criteria, including employees aged 20 years or older who were currently working in the hospitality and tourism industry. Invitations were sent only to qualified panel members, and each participant completed the survey only once. To ensure demographic representativeness, stratified random sampling was applied based on the gender and age distribution of Korea’s hospitality and tourism workforce (Statistics Korea, 2022). Prior to participation, respondents were informed of the study purpose and procedures and their rights, including the right to withdraw at any time. Data were collected only after informed consent was obtained, ensuring ethical compliance and protection of personal information.

The study adopted a three-wave time-lagged design with one-month intervals between measurement waves to introduce temporal separation among key variables and strengthen causal inference. Prior research indicates that a one-month interval is adequate for detecting changes in employees’ work experiences and psychological states while reducing recall bias (Di Blasi et al., 2022; Fuller et al., 2016). Similar longitudinal studies examining innovative work behavior have also adopted one-month intervals, supporting the appropriateness of this temporal design (Riaz et al., 2018; Zia et al., 2024). This design also allows the psychological and behavioral processes of interest to unfold naturally within organizational contexts (Mao et al., 2024).

In the first wave, questionnaires measuring AI self-efficacy were distributed to 950 employees, resulting in 620 valid responses (response rate = 65.3%). In the second wave, the same 620 employees were invited to complete a survey assessing their work engagement and schedule i-deals, and 420 valid responses were obtained, resulting in a follow-up rate of 44.2% (420/950). In the third wave, the remaining 420 employees were surveyed regarding their innovative work behavior, yielding 300 valid responses. Consequently, the final sample represented a retention rate of 31.6% (300/950) (see Appendix A).

Of the 300 respondents included in the final analysis, 174 (58.0%) were male and 126 (42.0%) were female, with a mean age of 44.77 years (SD = 10.10). The distribution of educational attainment was as follows: 34 respondents (11.3%) had completed secondary education or below, 57 (19.0%) held a college diploma, 182 (60.7%) held a bachelor’s degree, 22 (7.3%) had a master’s degree, and 5 (1.7%) had a doctoral degree.

Because this study employed a three-wave time-lagged design, some sample attrition occurred across waves. Following the procedure recommended by Goodman and Blum (1996), attrition analyses were conducted to examine the presence of non-random sampling. Logistic regression analyses indicated that Wave 1 AI self-efficacy and demographic variables did not systematically predict attrition. Comparisons between retained participants and those who dropped out showed no significant differences in AI self-efficacy or education, although retained participants were somewhat older and differed slightly in gender composition. Age and gender were included as control variables in all analyses. In addition, a sensitivity analysis using inverse probability weighting was conducted following the attrition correction framework proposed by Wooldridge (2007). The IPW-adjusted estimates were broadly consistent with the primary results. Overall, these findings suggest that differential attrition on observed variables was limited and that the results are unlikely to be substantially biased by subject attrition.

### 3.2. Measurement

All scales were adapted from previously validated English-language measures and translated into Korean using Brislin’s (1980) translation-back translation procedure. The results confirmed that the Korean items accurately reflected the meanings of the original scales. All constructs in this study were measured using three-item short-form versions of established scales to reduce respondent burden while maintaining response quality in the multi-wave survey design (Stanton et al., 2002). All variables were measured on a 5-point Likert scale (1 = Not at all, 5 = Very much). Measurement items are presented in Appendix B.

#### 3.2.1. AI Self-Efficacy

Employees’ AI self-efficacy was measured using a scale adapted from the self-efficacy dimension of Spreitzer’s (1995) psychological empowerment instrument. Consistent with domain-specific self-efficacy theory (Bandura, 2006) and prior organizational research (e.g., B.-J. Kim et al., 2024), this study conceptualizes AI self-efficacy as a unidimensional construct reflecting employees’ efficacy beliefs regarding their capability to use AI tools for job-related tasks, rather than technical AI proficiency. In contextualizing the original items for AI-related work situations, the procedures recommended by B.-J. Kim et al. (2024) were followed to ensure that the adapted items accurately reflected employees’ confidence in using artificial intelligence technologies. The final scale consisted of three items. An example item is “I am self-assured about my capabilities in using artificial intelligence technology to perform my work activities well.”

#### 3.2.2. Work Engagement

Work engagement was measured using a three-item scale developed by Schaufeli et al. (2017). An example item is “I am enthusiastic about my job.” Cronbach’s α for this scale was 0.88.

#### 3.2.3. Schedule I-Deals

Schedule i-deals were measured using a three-item scale developed by Velasco Vizcaíno et al. (2023). An example item is “My supervisor considers my personal needs when making my work schedule.” Cronbach’s α for this scale was 0.86.

#### 3.2.4. Innovative Work Behavior

Innovative work behavior was measured using a three-item scale developed by Schuh et al. (2018). An example item is “I am searching out new working methods, techniques, or instruments.” Cronbach’s α for this scale was 0.87.

#### 3.2.5. Control Variables

In selecting control variables, this study followed the guidelines proposed by Bernerth and Aguinis (2016). Additionally, the empirical findings of Newman et al. (2018) indicate that gender, age, and final level of education are significantly related to the dependent variable, namely innovative work behavior. Accordingly, these three demographic characteristics were included as control variables in the subsequent statistical analyses.

### 3.3. Assessment of Common Method Variance

To minimize the potential impact of common method bias, this study first implemented procedural remedies through a three-wave, time-lagged research design. Harman’s single-factor test was subsequently conducted to assess potential common method bias. The analysis showed that the first unrotated factor accounted for only 27% of the total variance. Two additional analyses were conducted. First, a common latent factor test (Podsakoff et al., 2003) showed good model fit (χ^2^ = 64.43, CFI = 0.99, TLI = 0.98, RMSEA = 0.04) with stable factor loadings. Second, the marker variable technique (Lindell & Whitney, 2001) indicated that focal path estimates remained virtually unchanged after controlling for the marker variable (maximum change = 1.48%). Together with the procedural controls, these results suggest that common method bias is unlikely to pose a serious threat to the validity of the study’s findings (Podsakoff et al., 2003).

### 3.4. Analytic Strategy

All data processing and statistical analyses were conducted using Stata 18.0 (StataCorp LLC, College Station, TX, USA). First, descriptive statistics and correlations among the key variables were examined, and the internal consistency of the measurement instruments was assessed using Cronbach’s α. Next, confirmatory factor analysis (CFA) was performed to evaluate the fit of the measurement model, and average variance extracted (AVE) and composite reliability (CR) were calculated to assess convergent and discriminant validity. For testing the hypotheses, hierarchical regression analyses were conducted, and mediation as well as moderated mediation effects were examined using bootstrapping procedures following the recommendations of Hayes (2018).

## 4. Results

### 4.1. Preliminary Analyses

Table 1 presents the descriptive statistics for all study variables, including means, standard deviations, correlation coefficients, and Cronbach’s α values. The results indicate that the observed correlations among the variables are generally consistent with the hypotheses of this study.

Table 2 reports the results of the CFA assessing the structural validity of the measurement model. The normed chi-square (χ^2^/df) is 1.12, which satisfies the recommended cutoff of <3.00 (Hair et al., 2010). Regarding the comparative fit indices, both the CFI and TLI are 0.99, exceeding the recommended threshold of 0.95. The RMSEA value of 0.02 satisfies not only the acceptable criterion of <0.08 but also the more stringent criterion of <0.05 (Hair et al., 2010). Furthermore, the hypothesized research model was evaluated against several alternative models. The comparative CFA results indicate that the proposed model provides the best fit and most adequately captures the structural characteristics of the data.

The AVE values for all latent variables exceeded 0.50 (min = 0.67), indicating adequate convergent validity (Hair et al., 2010). Moreover, the correlation coefficients among the constructs were all lower than the square root of each construct’s AVE, thereby establishing discriminant validity (Hair et al., 2010). In addition, all CR values were greater than 0.70 (min = 0.86), further confirming construct reliability.

Data normality was assessed using skewness and kurtosis. The results showed that skewness ranged from −0.95 to −0.30, which is within the acceptable ±2 threshold (Ryu, 2011). Kurtosis ranged from 3.03 to 4.26, below the ±7 criterion (Ryu, 2011). Overall, the data distribution approximated normality, fulfilling the basic assumptions for multivariate statistical analyses and providing a basis for subsequent model testing.

### 4.2. Hypotheses Testing

Hypotheses 1 and 3 were tested using hierarchical regression analysis, whereas Hypothesis 2 was examined through a bootstrapping procedure. To test Hypothesis 1, which proposes that AI self-efficacy has a positive relationship with innovative work behavior, Model 4 of Table 3 shows that AI self-efficacy has a significant positive effect on innovative work behavior (*β* = 0.34, *p* < 0.001). Furthermore, Model 4 explained significantly more variance than Model 3 (ΔR^2^ = 0.10, *p* < 0.001). Therefore, Hypothesis 1 was supported.

To test Hypothesis 2, the mediating effect was examined using 10,000 bootstrap resamples. As shown in Table 4, the 95% confidence interval for the indirect effect [0.03, 0.18] excluded zero, indicating a statistically significant indirect effect. Although the effect size was modest, the findings support an indirect pathway through work engagement. Therefore, Hypothesis 2 was supported.

To test Hypothesis 3 and reduce multicollinearity, the relevant variables were mean-centered following the recommendations of Aiken and West (1991). Subsequently, an interaction term between work engagement and schedule i-deals was created to examine the moderating effect. As shown in Model 6 of Table 3, the coefficient of the interaction term was positive and significant (*β* = 0.22, *p* < 0.001). Furthermore, the explanatory power of the model increased significantly compared with Model 5. Thus, these results provide support for Hypothesis 3.

**Figure 2:**
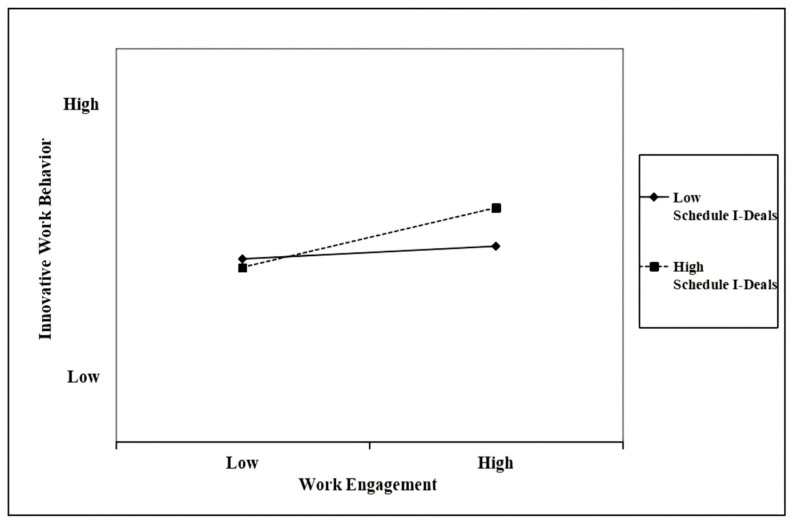
The moderating effect of schedule i-deals on the relationship between work engagement and innovative work behavior. Source: Authors’ own work.

To further verify the moderating effect, a simple slope analysis was conducted following the procedure of Aiken and West (1991). Figure 2 illustrates the conceptual regression lines for work engagement predicting innovative work behavior at low (mean − 1 SD) and high (mean + 1 SD) levels of the moderator, schedule i-deals. The results showed that when schedule i-deals were low, the effect of work engagement on innovative work behavior was not significant (b = 0.12, *p =* 0.067, n.s.). However, when schedule i-deals were high, the relationship was significant (b = 0.43, *p* < 0.001). These results offer clear evidence of the moderating effects proposed in this study.

Finally, to test the moderated mediation effect proposed in Hypothesis 4, this study conducted the analysis in Stata using 10,000 bootstrap resamples and bias-corrected 95% confidence intervals. This procedure is equivalent to Model 14 of Hayes’ PROCESS macro (Hayes, 2013). As shown in Table 5, the indirect effect of AI self-efficacy on innovative work behavior through work engagement was significant. However, its magnitude differed depending on the level of schedule i-deals. When schedule i-deals were high (mean + 1 SD), the indirect effect was 0.08, whereas when they were low (mean − 1 SD), the effect decreased to 0.02. The pattern showing a stronger indirect effect at higher levels of schedule i-deals provides clear evidence of a moderated mediation effect and supports Hypothesis 4 (Preacher et al., 2007).

## 5. Discussion

### 5.1. Summary

From the JD-R perspective, the study examined how AI self-efficacy influences innovative work behavior using a three-wave time-lagged design with employees in the hospitality and tourism service sectors. First, the findings show that AI self-efficacy, as a personal resource, promotes innovative work behavior. Employees with high AI self-efficacy feel greater competence and control when using technology and view AI not as a mere object of dependence but as an enabling tool that enhances their work. This personal resource helps them respond more effectively to increasingly complex and dynamic job demands and encourages them to engage in innovative actions by leveraging AI support.

Second, the findings indicate that work engagement mediates the relationship between AI self-efficacy and innovative work behavior. When employees believe they can use AI effectively, their sense of competence and motivation increases, and this heightened level of engagement subsequently translates into innovative actions. Furthermore, this mediating effect depends on the job resource of schedule i-deals. When organizations provide employees with sufficient temporal autonomy, the engagement driven by AI self-efficacy is more likely to be translated into innovative work behavior.

In summary, this study suggests that innovative work behavior in the AI era arises from the interaction between personal and job resources. These findings contribute to a human-centered perspective to the ongoing debate over whether AI enhances or diminishes creativity (Yin et al., 2024) and offer important implications for managers in the service sector. Specifically, strengthening employees’ AI self-efficacy, together with establishing flexible work arrangements and supportive organizational environments, is essential for realizing the innovative potential enabled by AI technologies.

### 5.2. Theoretical Contributions

First, this study provides new insights into employees’ innovative work behavior in the AI era from the perspective of individual agency. Whereas prior studies have mainly focused on the effects of AI technology itself on employees’ creativity (e.g., Jeong, 2025), this study highlights employees’ AI self-efficacy in the process of using AI technologies. By demonstrating that AI self-efficacy fosters innovative work behavior, the study extends this emerging concept to high-contact service sectors such as the hospitality and tourism industry, thereby responding to the call by Y.-Y. Wang and Chuang (2024) for a deeper exploration of how AI self-efficacy shapes employee motivation and behavior. The findings show that employees are not passive recipients of technology. Rather, their beliefs and confidence in AI directly influence their behavioral patterns. This offers a theoretical foundation for future research examining the relationship between technology and employee behavior from the perspective of individual differences.

Second, the findings extend the applicability of the JD-R model by conceptualizing AI self-efficacy as a personal resource and AI use as a new job demand. The findings demonstrate that when personal and job resources are jointly considered within AI-supported work contexts, employees’ innovative work behavior is enhanced, thereby strengthening the explanatory power of the JD-R model in AI environments. Moreover, these results offer a novel theoretical lens for interpreting the ongoing debate on whether AI facilitates or inhibits innovative work behavior, an issue on which prior studies have not yet reached consensus (Yin et al., 2024).

Third, by introducing work engagement as a mediating mechanism, this study explains how AI self-efficacy translates into innovative work behavior, thereby further validating the motivational pathway proposed by the JD-R model. The results show that employees with strong confidence in their AI capabilities exhibit higher levels of enthusiasm and absorption in their work, which in turn leads to voluntary innovative work behavior. This finding deepens the understanding of the relationship between AI self-efficacy and innovative work behavior and provides an academic response to the growing scholarly interest in exploring the antecedents of work engagement and its influence on key work outcomes (Ali et al., 2022).

Fourth, this study introduces schedule i-deals as a job resource and demonstrates their moderating effect on the relationship between work engagement and innovative work behavior, as well as their moderated mediation effect within the overall mechanism. These findings provide theoretical support for the JD-R model by showing that job resources such as temporal flexibility can function as moderators. Moreover, they reveal that the positive influence of personal resources on employee behavior through the motivational pathway is not fixed but varies depending on the level of available job resources. The findings provide new empirical evidence for the resource gain cycle proposed in the JD-R framework (Bakker et al., 2023) and contribute to broadening the scope of research on schedule i-deals.

### 5.3. Practical Implications

This study offers several practical recommendations for management practices in service industries such as hospitality and tourism industry. First, it is essential to strengthen employees’ AI self-efficacy. The findings indicate that fostering innovative work behavior does not rely solely on technological advancement. Employees’ confidence in using AI also plays a critical role. Therefore, as organizations pursue digitalization and intelligent transformation, they must also enhance AI-related training for employees. Training programs should go beyond introducing technical functions and incorporate case-based examples relevant to frontline operational contexts (Han et al., 2025). For instance, clearly demonstrating how AI assists frontline staff in identifying customer preferences or supports scheduling optimization in room management can help employees recognize the practical value of AI more clearly. Enhancing employees’ AI proficiency and confidence not only reduces anxiety about technological replacement but also enables them to view AI as a meaningful resource that improves service quality and creates customer value.

Second, the findings highlight the mediating role of work engagement in the relationship between employees’ AI self-efficacy and their innovative work behavior. Work engagement represents a positive psychological state at work (Agarwal, 2014), characterized by employees’ enthusiasm and energetic involvement in their tasks. Organizations should therefore implement managerial practices that enable employees to use and experience AI more positively in their work. For instance, granting employees autonomy to choose and utilize AI tools suited to their tasks can strengthen their sense of ownership and enhance their enthusiasm toward their work (Ali et al., 2022).

Third, when employees use AI to improve work processes or propose innovative ideas, leaders should provide timely recognition and supportive feedback to alleviate concerns about innovation-related risks. For example, leaders may publicly acknowledge employees’ attempts to apply AI during meetings, and when employees encounter difficulties with using AI, leaders should provide technical guidance or offer the necessary resources. Such forms of support help employees maintain a high level of work engagement and strengthen their willingness to continuously explore the innovative opportunities that AI can provide.

Fourth, the findings highlight the critical role of schedule i-deals in moderating the relationship between work engagement and innovative work behavior. Employees who are granted greater flexibility in negotiating their work hours can more effectively turn engagement into innovative work behavior. As the boundaries between work and personal life continue to blur, schedule i-deals have become increasingly common in organizations and have gained attention for allowing employees more autonomy in adjusting their work schedules (Taser et al., 2022). Although work schedules in the hospitality and tourism sectors are often highly structured, managers still retain considerable discretion over how time is allocated (Kelly et al., 2020). Therefore, managers should pay closer attention to employees’ individual needs and, within feasible limits, offer options such as flexible shifts, schedule adjustments, or preferred working hours (Shifrin & Michel, 2022). Such forms of support not only help employees sustain their level of work engagement but also foster the innovative potential needed to enhance service processes and improve customer experience.

### 5.4. Research Limitations and Future Research Directions

Although this study advances understanding of the impact of AI self-efficacy on innovative work behavior, several limitations should be acknowledged, offering important directions for future research. First, self-efficacy is inherently domain- and context-specific and may therefore manifest differently across technological environments and occupational roles (Bandura, 2006). In this study, AI self-efficacy refers to employees’ beliefs in their ability to use AI, reflecting their role as users of AI systems in the hospitality and tourism context. Future research may extend this work by examining this belief across different AI applications and levels of technological complexity to further clarify its boundary conditions. Furthermore, as AI self-efficacy may encompass multiple dimensions in more technically demanding contexts, future research should develop more comprehensive and multidimensional measures to assess its distinct facets and their differential effects on work outcomes.

Second, because the data were collected primarily through surveys, the ability to draw strong causal inferences remains limited. Although the three-wave time-lagged design grounded in the JD-R model helps reduce common method concerns, schedule i-deals and work engagement were measured at the same time point, which constrains the establishment of clear temporal precedence. Attrition is an additional concern in multi-wave research. Although diagnostic analyses (Goodman & Blum, 1996) and supplementary inverse probability weighting sensitivity tests (Wooldridge, 2007) suggested that the pattern of results remained substantively similar after adjusting for observed attrition, the possibility of attrition related to unobserved characteristics cannot be fully excluded. Accordingly, the findings should be interpreted with appropriate caution. Future research could further strengthen causal inference by adopting experimental designs or more fine-grained longitudinal approaches. For instance, interventions aimed at enhancing AI self-efficacy could be implemented to examine subsequent innovative work behavior. Alternatively, a four-wave design measuring study variables at distinct time points would allow clearer tests of temporal ordering.

Third, this study relied primarily on self-reported data, which may introduce a certain degree of common method bias. Although prior research suggests that self-reported measures do not substantially undermine data validity (Chan, 2010; W. Wang et al., 2024), and although Harman’s single-factor test, common latent factor analysis, and marker variable analysis collectively indicate that common method bias is unlikely to be the primary driver of the findings, the use of self-report questionnaires still carries inherent limitations. In particular, responses may be influenced by social desirability or respondents’ subjective perceptions (Durmaz et al., 2020). Future research could therefore benefit from incorporating multiple data sources. For instance, combining supervisors’ behavioral evaluations or colleagues’ observations of interpersonal interactions could help reduce bias stemming from a single-source design.

Fourth, all constructs in this study were measured using established three-item scales, and the original item wording was retained to preserve the validated scale structures. Although these measures demonstrated satisfactory reliability and validity, three indicators represent the minimum typically recommended for latent variable modeling and may limit flexibility in assessing item-level misspecification. Three-item scales may also provide more limited content coverage than scales with more items. In addition, the absence of reverse-coded items may represent a potential measurement constraint, as responses may be influenced by acquiescence tendencies. Future research may therefore benefit from employing more comprehensive measures and incorporating reverse-coded items to further strengthen measurement design.

Fifth, the data used in this study were collected primarily from employees working in the Korean hospitality and tourism industry. Although the translation and localization process carefully accounted for cultural characteristics to ensure accurate adaptation from English to Korean, the findings may still reflect contextual influences inherent in local values and organizational norms. For example, cultural attributes commonly observed in Korean workplaces—such as high power distance and collectivism (Ding & Hong, 2025)—may shape how employees develop AI self-efficacy and, consequently, influence the pathways leading to innovative work behavior. To enhance the generalizability and cross-context applicability of the conclusions, future studies should broaden their sampling frame. Specifically, cross-cultural comparisons (e.g., Korea vs. low power distance cultures) could examine whether the findings generalize across contexts. This may involve including participants from countries with differing cultural backgrounds, as well as validating the proposed mechanisms across diverse industries such as manufacturing, finance, and high-tech sectors, thereby providing a more comprehensive assessment of the robustness of the observed relationships.

Sixth, this study focused exclusively on schedule i-deals—that is, individualized agreements through which employees negotiate work-time adjustments with their supervisors—because employees in the hospitality and tourism industry typically operate under long working hours and rigid scheduling structures. Other forms of i-deals, such as developmental, task-related, or reward-based i-deals, were not examined. However, different categories of i-deals may influence employees’ work engagement and innovative work behavior through distinct mechanisms. Therefore, future studies should incorporate multiple types of i-deals to more comprehensively explain how individualized arrangements shape employees’ attitudes and behavioral outcomes.

Finally, grounded in the JD-R framework, this study conceptualized AI self-efficacy as a personal resource and schedule i-deals as a job resource to explain their joint effects on innovative work behavior. Nonetheless, this mechanism may also be interpreted through alternative theoretical lenses. Future research could employ Self-Determination Theory (Deci & Ryan, 2008) to clarify these relationships from the perspective of satisfying fundamental psychological needs such as competence and autonomy. AI self-efficacy may enhance employees’ perceived competence when using AI, while the flexibility granted through schedule i-deals can strengthen autonomy, thereby fostering innovative work behavior. Subsequent research integrating self-determination theory could provide deeper insights into these motivational pathways and further elucidate how innovative work behavior emerges in AI-driven work contexts.

## Figures and Tables

**Table 1:** Means, Standard Deviations, Correlations, and Reliabilities.

Variables	Mean	SD	1	2	3	4	5	6	7
1. Gender	0.42	0.49							
2. Age	44.77	10.10	−0.36 ***						
3. Education	2.69	0.83	−0.14 *	−0.15 *					
4. AI self-efficacy	3.16	0.83	−0.08	−0.17 **	0.15 *	(0.88)			
5. Work engagement	3.30	0.76	−0.07	0.16 **	−0.05	0.21 ***	(0.88)		
6. Schedule i-deals	3.54	0.81	−0.09	−0.04	0.09	0.12 *	0.41 ***	(0.86)	
7. Innovative work behavior	3.29	0.77	−0.18 **	0.10	0.13 **	0.33 ***	0.33 ***	0.19 **	(0.87)

Notes. *n* = 300; * *p* < 0.05, ** *p* < 0.01, *** *p* < 0.001 (two-tailed test). The values in parentheses denote Cronbach’s α. Gender: 0 = male, 1 = female. Age: age in years. Education: final level of education: 1 = high school or below, 2 = college graduate, 3 = four-year university graduate, 4 = master’s degree, 5 = Ph.D. holder. Source: Authors’ own work.

**Table 2:** Confirmatory Factor Analysis Results.

Model	χ^2^ (df)	CFI	TLI	RMSEA	Δχ^2^ (Δdf)
Research model (four factor)	80.57 (72) ***	0.99	0.99	0.02	
Alternative model ^1^ (three factor) ^1^	625.39 (78) ***	0.74	0.66	0.15	544.81 (6) ***
Alternative model ^2^ (two factor) ^2^	1030.54 (83) ***	0.54	0.44	0.20	949.97 (11) ***
Alternative model ^3^ (one factor) ^3^	1426.44 (87) ***	0.35	0.24	0.23	1345.86 (15) ***

Notes. *n* = 300; *** *p* < 0.001. ^1^ Three-factor model with AI self-efficacy and work engagement on the same factor. ^2^ Two-factor model with AI self-efficacy, work engagement, and schedule i-deals on the same factor. ^3^ One-factor model with AI self-efficacy, work engagement, schedule i-deals, and innovative work behavior on the same factor. CFI = comparative fit index; TLI = Tucker–Lewis index; RMSEA = root mean square error of approximation. Source: Authors’ own work.

**Table 3:** Results of Hierarchical Multiple Regression.

Variable	Work Engagement		Innovative Work Behavior			
	Model 1	Model 2	Model 3	Model 4	Model 5	Model 6
Gender	−0.03	0.01	−0.14 *	−0.09	−0.09	−0.09
Age	0.14 *	0.20 **	0.07	0.14 *	0.09	0.08
Education	−0.03	−0.06	0.12	0.08	0.10	0.10 *
AI self-efficacy		0.25 ***		0.34 ***	0.27 ***	0.26 ***
Work engagement					0.25 ***	0.27 ***
Schedule i-deals						0.10
WE × SID						0.22 ***
R^2^	0.03	0.08	0.05	0.15	0.21	0.26
ΔR^2^		0.05		0.10	0.06	0.05
adj. R^2^	0.02	0.07	0.04	0.14	0.20	0.24
F	2.62	6.78 ***	4.75 **	13.25 ***	15.64 ***	14.32 ***
Finc		18.79 ***		37.04 ***	21.49 ***	8.92 ***

Notes. *n* = 300; * *p* < 0.05, ** *p* < 0.01, *** *p* < 0.001 (two-tailed test). The results are standardized regression coefficients. Gender: 0 = male, 1 = female. Age: age in years. Education: final level of education: 1 = high school or below, 2 = college graduate, 3 = four-year university graduate, 4 = master’s degree, 5 = Ph.D. holder. WE = work engagement; SID = schedule i-deals. Source: Authors’ own work.

**Table 4:** Results of Bootstrapped Indirect Effect Test.

	Dependent Variable: Innovative Work Behavior			
Mediator	Indirect Effect	SE	95% CI	
			LLCI	ULCI
Work engagement	0.09	0.00	0.03	0.18

Notes. *n* = 300; number of bootstrapping iterations = 10,000. Abbreviations: SE, standard error; CI, confidence interval; LLCI, lower limit of confidence interval; ULCI, upper limit of confidence interval. Source: Authors’ own work.

**Table 5:** Results of the Conditional Bootstrapped Indirect Effects Test.

		Dependent Variable: Innovative Work Behavior			
Moderator	Level of Moderator	Indirect Effect	SE	95% CI	
				LLCI	ULCI
Schedule i-deals	Low (Mean − 1 SD)	0.02	0.02	0.00	0.07
	High (Mean + 1 SD)	0.08	0.03	0.03	0.15

Notes. *n* = 300; number of bootstrapping iterations = 10,000. SD = standard deviation; SE = standard error; LLCI = lower limit of confidence interval; ULCI = upper limit of confidence interval. Source: Authors’ own work.

**Figure A1:**
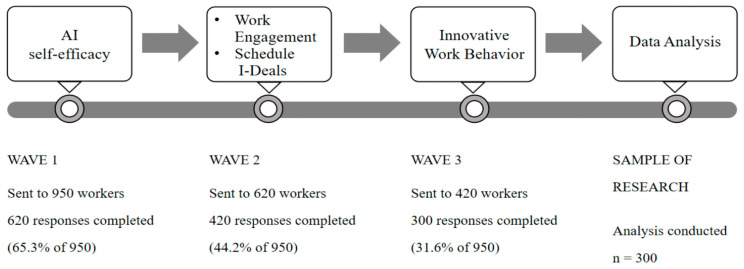
Sampling procedure and data collection across three waves.

## Data Availability

The raw data supporting the conclusions of this article will be made available by the authors, without undue reservation, to any qualified researcher.

## Appendix B. Measurement

**AI self-efficacy (α = 0.88) (Spreitzer, 1995; B.-J. Kim et al., 2024)**

1. I am confident about my ability to utilize artificial intelligence technology appropriately in my work.
2. I am self-assured about my capabilities to use artificial intelligence technology to perform my work activities well.
3. I have mastered the skills necessary for the use of artificial intelligence technology in my job.

**Work engagement (α = 0.88) (Schaufeli et al., 2017)**

1. At my work, I feel bursting with energy.
2. I am enthusiastic about my job.
3. I am immersed in my work.

**Schedule idiosyncratic deals (α = 0.86) (Velasco Vizcaíno et al., 2023)**

1. My supervisor considers my personal needs when making my work schedule.
2. At my request, my supervisor has accommodated my off-the-job demands when assigning my work hours.
3. Outside of formal leave and sick time, my supervisor has allowed me to take time off to attend to non-work-related issues.

**Innovative work behavior (α = 0.87) (Schuh et al., 2018)**

1. I am searching out new working methods, techniques, or instruments.
2. I am mobilizing support for innovative ideas.
3. I am transforming innovative ideas into useful applications.
